# Genome Sequence of a Plant-Pathogenic Bacterium, “Candidatus Phytoplasma asteris” Strain TW1

**DOI:** 10.1128/MRA.01109-18

**Published:** 2018-09-27

**Authors:** Jennifer R. Town, Tyler Wist, Edel Perez-Lopez, Chrystel Y. Olivier, Tim J. Dumonceaux

**Affiliations:** a Agriculture and Agri-Food Canada, Saskatoon Research and Development Centre, Saskatoon, Saskatchewan, Canada; b Department of Biology, University of Saskatchewan, Saskatoon, Saskatchewan, Canada; University of Arizona

## Abstract

A draft genome sequence is presented for a strain of “Candidatus Phytoplasma asteris” affecting canola plants in Saskatoon, Canada. This phytopathogenic bacterium was determined to be a 16SrI strain and features 16S rRNA-encoding gene sequence heterogeneity.

## ANNOUNCEMENT

Plants infected by insect-vectored phytoplasma (“Candidatus Phytoplasma spp.”) feature a pathological morphology that includes virescence (abnormal green coloration) and phyllody (development of leaf-like structures) in floral tissues (1). In cultivated plants, such as canola (Brassica napus L.), this altered plant development results in abnormal inflorescence morphology with a reduced seed set, decreasing the value of the crop (2).

Highly symptomatic B. napus plants were observed in Saskatoon, Canada (52.155°N, 106.57°W), in July 2017. Inflorescence tissue from a single plant (100 mg [wet weight]) was used for DNA extraction using a Qiagen plant DNA minikit. Genomic DNA (1 µg) was used as input for bacterial DNA selection (NEBNext microbiome enrichment kit; New England BioLabs). The DNA remaining after selection was sheared by sonication using a Bioruptor 300 (Diagenode) on high power, with 30 cycles (30 s on/30 s off) at 4°C. Fragmented DNA (average size, 303 bp) was prepared for sequencing using the NEBNext Ultra DNA library prep kit for Illumina (New England BioLabs) and then sequenced on the MiSeq platform (600 cycles). This generated 1.24 million paired reads, which were mapped to publicly available aster yellows phytoplasma (AYp) genomes (“*Ca*. Phytoplasma asteris” CYP, GenBank accession no. JSWH00000000, and “*Ca*. Phytoplasma asteris” OY-M, GenBank accession no. NC_005303) using Bowtie 2 version 2.3.3.1 (3). This retrieved 81,266 Illumina reads, which provided a fragmented genome (661 scaffolds) on assembly using SOAPdenovo2 version 2.01 (4). To improve the assembly, further sequencing was performed using the Oxford Nanopore sequencing platform (ONT, London, UK). Total genomic DNA was purified from inflorescence tissue using a column-free protocol (5), and long fragments were size selected using AMPure XP beads (Beckman Coulter) at 0.45 (vol/vol). Microbial DNA was selected as described above, and 356 ng was prepared for sequencing with a native barcoding kit 1D (ONT) and then sequenced on a nanopore flow cell R9.4. ONT sequencing provided 94,981 reads that ranged in length from 101 to 78,510 bp (mean, 1,743 bp). The mean quality score of the reads was 11.4 (range, 4.91 to 15.95). Illumina reads that mapped to AYp genomes (81,266 reads) were combined with unfiltered ONT reads for coassembly with Unicycler version 0.4.4 (6), with default parameters. This assembly resulted in 11 scaffolds, of which 6 scaffolds corresponded to Brassica napus DNA and 5 scaffolds were determined to derive from “*Ca*. Phytoplasma asteris” by BLAST (7). The 5 scaffolds corresponding to “*Ca*. Phytoplasma asteris” ranged in length from 4,092 to 382,354 bp and provided a draft genome sequence (8) with an average coverage of 146×. The finished genome sequence was annotated using the Prokaryotic Genome Annotation Pipeline version 3.1 (9).

The assembled genome of “*Ca*. Phytoplasma asteris” strain TW1 contained 743,598 bp (28.3% G+C content). A total of 741 genes were annotated in the genomic sequence, including 457 coding genes, 36 RNA-encoding genes, and 248 pseudogenes. The genome sequence contained two distinct 16S rRNA-encoding genes that were located on a single scaffold. The restriction digestion pattern of one of these genes corresponded to 16SrI-B (*F* = 1.00) using the *i*PhyClassifier (10), while the other copy corresponded to 16SrI-A (*F* = 0.97). The single-copy phytoplasma taxonomic marker *cpn60* (11) typed as I-IB (*F* = 1.00) using random fragment length polymorphism (RFLP) analysis (12). Other taxonomic markers, including *secY*, *secA*, *nusA,* and *rp*, were also found in a single copy in the genome sequence. This indicates that “*Ca*. Phytoplasma asteris” TW1 is a 16SrI strain featuring 16S rRNA-encoding gene heterogeneity (13).

### Data availability.

The sequence data for this complete genome have been deposited at DDBJ/EMBL/GenBank under the accession no. QGKT00000000. Raw sequence reads (ONT and Illumina) have been deposited to the NCBI Sequence Read Archive with accession no. SRP154591.
